# Magnetic Resonance T1w/T2w Ratio in the Putamen and Cerebellum as a Marker of Cognitive Impairment in MSA: a Longitudinal Study

**DOI:** 10.1007/s12311-022-01455-8

**Published:** 2022-08-19

**Authors:** Sofia Cuoco, Sara Ponticorvo, Rossella Bisogno, Renzo Manara, Fabrizio Esposito, Gianfranco Di Salle, Francesco Di Salle, Marianna Amboni, Roberto Erro, Marina Picillo, Paolo Barone, Maria Teresa Pellecchia

**Affiliations:** 1 Center for Neurodegenerative Diseases (CEMAND), Department of Medicine, Surgery and Dentistry, Neuroscience Section, University of Salerno, 84131 Salerno, Italy; 2 Neuroradiology Unit, Department of Neurosciences, University of Padua, 35128 Padua, Italy; 3 Department of Advanced Medical and Surgical Sciences, University of Campania “Luigi Vanvitelli, Napoli, Italy; 4 Scuola Superiore Di Studi Universitari E Perfezionamento Sant’Anna, Classe Di Scienze Sperimentali, Pisa, Italy

**Keywords:** Cerebellum, Cognitive impairment, Magnetic resonance imaging, Multiple system atrophy, Putamen, T1w/T2w

## Abstract

The exact pathophysiology of cognitive impairment in multiple system atrophy (MSA) is unclear. In our longitudinal study, we aimed to analyze (I) the relationships between cognitive functions and some subcortical structures, such as putamen and cerebellum assessed by voxel-based morphometry (VBM) and T1-weighted/T2-weighted (T1w/T2w) ratio, and (II) the neuroimaging predictors of the progression of cognitive deficits. Twenty-six patients with MSA underwent a comprehensive neuropsychological battery, motor examination, and brain MRI at baseline (*T*_0_) and 1-year follow-up (*T*_1_). Patients were then divided according to cognitive status into MSA with normal cognition (MSA-NC) and MSA with mild cognitive impairment (MCI). At *T*_1_, we divided the sample according to worsening/non worsening of cognitive status compared to baseline evaluation. Logistic regression analysis showed that age (*β* =  − 9.45, *p* = .02) and T1w/T2w value in the left putamen (*β* = 230.64, *p* = .01) were significant predictors of global cognitive status at *T*_0_, explaining 65% of the variance. Logistic regression analysis showed that ∆-values of WM density in the cerebellum/brainstem (*β* = 2188.70, *p* = .02) significantly predicted cognitive worsening at *T*_1_, explaining 64% of the variance. Our results suggest a role for the putamen and cerebellum in the cognitive changes of MSA, probably due to their connections with the cortex. The putaminal T1w/T2w ratio may deserve further studies as a marker of cognitive impairment in MSA.

**Supplementary Information:**

The online version contains supplementary material available at 10.1007/s12311-022-01455-8.

## Introduction

Multiple system atrophy (MSA) is a sporadic, progressive neurodegenerative disease characterized by parkinsonian features, cerebellar ataxia, autonomic failure, urogenital dysfunction and corticospinal disorders. The two subtypes of MSA are MSA with predominant parkinsonism (MSA-P) and MSA with predominant cerebellar ataxia (MSA-C) [1].

Cognitive impairment, characterized by prevalent executive dysfunctions, is an integral feature of the disease, and it presents largely variable frequency among studies (between 33 and 83%) [2]. Kim et al. [3] demonstrated that cognitive dysfunction significantly correlated with thinning in the neocortex, cerebellum, and striatum in MSA-P, whereas Lee et al. [4] found that the severity of atrophic changes in the bilateral thalamus, the left cerebellum, and the left pericalcarine gyrus were significantly correlated with attentional, executive and visuospatial dysfunctions. In MSA-P, Caso et al. [5] found a correlation between cortical thinning in temporal regions and both global cognitive status and memory impairment. Furthermore, the frontal and temporal regions, caudate and hippocampus regional atrophy were related with memory scores in MSA [6].

On the other hand, a subcortical pathogenesis of cognitive impairment has been proposed for MSA, since MSA patients with cognitive impairment showed only focal volume reduction in the left dorsolateral prefrontal cortex compared with MSA with normal cognition.

In a previous study [7], investigating brain tissue integrity by means of voxel-based morphometry (VBM) and T1-weighted/T2-weighted (T1w/T2w) maps, a whole-brain voxel-level analysis revealed reduced volume in bilateral putamen and cerebellar gray matter (GM), reduced white matter (WM) volume in the cerebellum and brainstem, and increased mean regional values of T1w/T2w in bilateral putamen in MSA patients compared to both Parkinson’s disease (PD) patients and healthy controls (HC). Specifically, the ratio of the signal intensity of the T1-weighted and T2-weighted (T1w / T2w) MRI images has been used as semi-quantitative measure for myelin content in gray matter and its advantage that images with high spatial resolution can be easily acquired without complex modeling, during routine clinical examination [7].

The exact pathophysiology of cognitive dysfunction in MSA remains unclear. Since a model of subcortical cognitive impairment has been proposed for MSA [8], we assessed the neuropsychological and MRI changes occurring in MSA patients in a longitudinal study, aiming to analyze (I) the relationships between cognitive functions and some subcortical structures, such as putamen and cerebellum assessed by VBM and T1w/T2w ratio, and (II) the neuroimaging predictors of the progression of cognitive deficits.

## Methods

### Patients

Twenty-six patients with a diagnosis of probable MSA, of which 14 with the parkinsonian variant (MSA-P) and 12 with the cerebellar variant (MSA-C), according to current criteria [1] were enrolled at the Center for Neurodegenerative Diseases of the University of Salerno between November 2015 and April 2019. All patients underwent a neuropsychological battery, motor test, and magnetic resonance imaging (MRI) at baseline (*T*_0_) and at follow-up (*T*_1_), 12 months later. The local Ethics Committee approved the study, and all patients signed informed consent.

### Clinical, Motor, and Neuropsychological Assessments

Demographic and clinical features, for the whole sample and the two subtypes, are listed in Table 1.

**Table 1:** Clinical, demographic, and neuropsychological features in MSA patient

	MSA Median (IQR) *N* = 26 (11 M/15F)	MSA-P Median (IQR) *N* = 14 (7 M/7F)	MSA-C Median (IQR) *N* = 12 (4 M/8F)	*p*
Age	59.00 (10.0)	61.50 (11.3)	57.50 (8.3)	0.27
Education	9.50 (7.0)	11.00 (7.3)	8.00 (6.8)	0.67
Disease duration	4.00 (3.3)	4.00 (2.8)	4.50 (4.3)	0.42
UMSARS-I	19.50 (5.3)	20.00 (10.3)	18.50 (4.5)	0.39
UMSARS-II	20.00 (7.3)	22.50 (9.5)	19.00 (4.3)	0.08
UMSARS-IV	2.00 (1)	3.00 (1)	2.00 (1)	0.26

*Abbreviations*: *IQR* interquartile range, *M* male, *MSA* multiple system atrophy, *N* number, *p p*-value, *UMSARS* Unified Multiple System Atrophy Rating Scale

Disease severity was assessed with the Unified MSA Rating Scale, part two (UMSARS-II).

Cognitive abilities were assessed at *T*_0_ and *T*_1_ with the Montreal Cognitive Assessment (MOCA), recall scores of the Rey auditory verbal learning test (15-RAWLT-recall), recall and copy of Rey Osterrieth figure (ROCF), short story test (Prosa Test), Trail Making Test-part A (TMT-A), Stroop Interference Test-error effect (SCWT), clock design test (CDT), verbal fluency test, constructional apraxia test (CA), and Benton orientation line test (BJLO) and sub-tests of E.N.P.A., such as words and non-words repetition tasks and auditory comprehension task (The percentage of complete tests are reported in Supplementary Material, S1).

Functional autonomy was evaluated with the Instrumental Activities of Daily Life (IADL) and with the Basic Activities of Daily Life (ADL).

Patients were divided according to cognitive status in MSA with normal cognition (MSA-NC) and MSA with mild cognitive impairment (MCI), including both patients with MCI-single domain (MSA-sd) and with MCI-multiple domain (MCI-md). Due to the lack of specific criteria for the diagnosis of MCI in MSA, it was diagnosed according to the MDS criteria for MCI in Parkinson’s disease [9]. At *T*_1_, we divided the sample according to worsening/nonworsening of cognitive status from *T*_0_ to *T*_1_.

### MRI Acquisition and Data Processing

All brain imaging data (at baseline as well as at follow-up) were acquired on the same 3 T MRI scanner (MAGNETOM Skyra, Siemens, Erlangen Germany) operated with a 20-channel head and neck coil. The imaging protocol consisted of a 3D anatomical T1-weighted (T1w) Magnetization Prepared RApid Gradient Echo (MPRAGE) sequence with repetition time (TR) = 2400 ms and echo time (TE) = 2.25 ms, spatial resolution = 1 × 1 × 1 mm^3^, matrix size = 256 × 256, anterior–posterior phase encoding direction, generalized autocalibrating partially parallel acquisitions (GRAPPA) factor of 2 in phase-encoding direction and a 3D T2-weighted (T2w) Sampling Perfection with Application optimized Contrast using different angle Evolutions (SPACE) sequence with TR = 3200, TE = 408 ms, variable flip angle, resolution = 1 × 1 × 1 mm^3^, matrix size = 256 × 256, anterior–posterior phase encoding direction, GRAPPA factor of 2 in phase-encoding [10].

T1w native space images of each subject and each time point (*T*_0_ and *T*_1_) were segmented into GM and WM in native space using SPM12 [11].

In order to obtain semi-quantitative maps markers of myelin content, T1w and T2w images were first corrected for intensity nonuniformity with the bias correction tool implemented in the unified segmentation [12] and available in SPM12. Then the T2w images were linearly registered to the T1w images using the FSL tool FLIRT [13] for estimating and applying a rigid-body affine transformation with 6 degrees of freedom and cubic spline interpolation [14]. T1w/T2w maps were obtained using FSLMATHS to divide the T1w volumes by the corresponding aligned T2w ones.

For the group-level analysis, in order to maximize both intra- and inter- subject spatial alignment, the two anatomical T1w volumes of each subject (*T*_0_ and *T*_1_) were combined into one T1w average volume (T1w-avg) using the intra-subject longitudinal diffeomorphic transformation as implemented in SPM12 [15]. Then, the single-subject tissue probabilistic maps (GM maps, WM maps, and T1w/T2w maps) were aligned to the corresponding T1w-avg volume with the nonlinear warping calculated in the longitudinal intra-subject alignment. Finally, all T1w-avg images were normalized to the MNI standard space with the DARTEL [16] procedure and the same deformation fields were used to spatially normalize the maps of interest. The resulting tissue (GM and WM) probabilistic maps were modulated by the Jacobian determinants of the deformations to account for local compression and expansion due to linear and non-linear transformation, and all the maps were smoothed with an isotropic Gaussian kernel of 6 mm full width at half maximum (FWHM).

Regional mean values of each parameter (GM, WM and T1w/T2w) at each time point were calculated in each cluster binary masks obtained in the voxel-level analysis of our previous study [7] performed on the baseline data. Particularly, regional cluster values in bilateral putamen, cerebellar GM, and cerebellar/brainstem WM were considered as region of interest in the present study.

### Statistical Analysis

Correlations between neuropsychological tests and MRI data at *T*_0_ were analyzed by Spearman’s correlation test. The Mann–Whitney’s *U* test was used to compare MRI parameters between patients with MCI and NC at *T*_0_. Significant results of exploratory analyses were corrected for multiple comparisons, where necessary. A logistic regression analysis with bootstrap method was performed with MCI or NC as the dependent variable, *p* < 0.05 deemed significant to evaluate a possible effect of MRI parameters on cognitive status at *T*_0_. In addition to the MRI parameters, the age of the subjects was also included as an independent variable.

Changes in neuropsychological scores and MRI data between *T*_1_ and *T*_0_ were calculated as differential delta scores [Delta = ∆ (*T*_1_ − *T*_0_)]. Spearman’s correlations between ∆-value of neuropsychological tests and ∆-MRI data were conducted. We also assessed the same correlations in worsening sub-group separately. The Mann–Whitney’s *U* test was used to compare ∆-MRI values between worsening and non worsening patients. Where necessary, we applied correction for multiple comparisons. A logistic regression analysis with Bootstrap method was performed with worsening or nonworsening as the dependent variable, *p* < 0.05 deemed significant, to evaluate a possible effect of ∆-MRI data on cognitive status. In addition to the ∆-MRI data, the age of the subjects was also included as an independent variable.

Finally, Spearman’s correlations between neuropsychological tests and MRI data at *T*_0_ and between neuropsychological changes and MRI changes, ∆ (*T*_1_-*T*_0_), were analyzed also in MSA-C and MSA-P, separately. Due to the small sample, Mann–Whitney’s test and logistic regression were not performed in MSA-C and MSA-P separately.

All statistical analyses were performed by SPSS-20 (SPSS Inc., Chicago, IL).

## Results

At *T*_0_, CA was directly related to GM density in the cerebellum/brainstem (rho = 0.43; *p* = 0.03), WM density in the cerebellum/brainstem (rho = 0.40; *p* = 0.04), and T1w/T2w (WM) in the cerebellum/brainstem (rho = 0.56; *p* = 0.01). There was a negative correlation between GM density in the right putamen and TMT-A (rho =  − 0.40; *p* = 0.04). After correction for multiple comparisons, only correlation between T1w/T2w (WM) in the cerebellum/brainstem and CA was retained (Table 2).

**Table 2:** Significant Spearman’s correlations between neuropsychological tests and MRI parameters at *T*_0_

Test	MRI parameter	Rho	*p*
CA	GM density in the cerebellum/brainstem	0.43	0.03
	WM density in the cerebellum/brainstem	0.40	0.04
	T1w/T2w (WM) in the cerebellum/brainstem	0.56	0.01*
TMT-A	GM density in the right putamen	− 0.40	0.04

^*^After multiple comparisons correction

*Abbreviations*: *CA* constructional apraxia test, *GM* gray matter, *MRI* magnetic resonance imaging, *p p*-value, *T1w/T2w*, mean regional values of T1-weighted/T2-weighted, *TMT-A* part A of Trail Making Test, *WM* white matter

We found no significant differences in MRI parameters between patients with MCI (*N* = 6) and NC (*N* = 20) (*p* > 0.05).

Logistic regression analysis showed that age (*β* =  − 9.45, *p* = 0.02) and T1w/T2w value in the Left putamen (*β* = 230.64, *p* = 0.01) were significant predictors of global cognitive status at *T*_0_, explaining 65% of the variance (*R*^2^ = 0.65).

At *T*_1_, we calculated changes in neuropsychological scores and MRI parameters between *T*_1_ and *T*_0_. After correcting for multiple comparisons, a positive correlation was found between ∆-values of the GM density right putamen and words repetition E.N.P.A. test (rho = 0.47; *p* = 0.02). There was a negative correlation between ∆-values of the T1w/T2w (WM) cerebellum/brainstem and TMT-A (rho =  − 0.55; *p* = 0.01) (Table 3). In the group of patients worsening at *T*_1,_ negative correlations were found between ∆-values of the GM density in the right putamen and TMT-A (rho =  − 0.50; *p* = 0.03), and between ∆-values of the GM density in the Left putamen and non-words repetitions E.N.P.A. test (rho =  − 0.48; *p* = 0.04). There was a positive correlation between ∆-values of the GM density in the right putamen and word repetition E.N.P.A. test (rho = 0.48; *p* = 0.04). Moreover, a trend towards significance was found for the following correlation: ∆-values of the T1w/T2w in the cerebellum/brainstem GM and CA (rho = 0.49; *p* = 0.05) (Table 3).

**Table 3:** Significant Spearman correlations between ∆-value of neuropsychological tests and MRI parameters

Sample	Test	MRI parameter	Rho	*p*
Whole sample	**∆-**word repetition E.N.P.A. test	∆-GM density right putamen	0.47	0.02*
	∆-TMT-A	∆-T1w/T2w (WM) cerebellum/brainstem	− 0.55	0.01*
Cognitively worsened at *T*_1_	∆-TMT-A	∆-GM density right putamen	− 0.50	0.03*
	∆-non-word repetitions E.N.P.A. test	∆-GM density left putamen	− 0.48	0.04*
	**∆-**word repetition E.N.P.A. test	∆-GM density right putamen	0.48	0.04*
	∆-CA	∆-T1w/T2w (GM) cerebellum/brainstem	0.49	0.05
	∆-15-RAWLT- immediate recall	∆-GM density right putamen	− 0.43	0.07
	∆- non-word repetition E.N.P.A. test	∆-GM density cerebellum/brainstem	− 0.43	0.08

Statistically significant differences are indicated in bold

*After multiple-comparison correction

*Abbreviations*: **∆** delta (*T*_1_–*T*_0_), *15-RAWLT*, Rey’s auditory 15-word learning test, *CA* constructional apraxia, *E.N.P.A.* neuropsychological examination for aphasia, *GM* gray matter, *MRI* magnetic resonance imaging, *p p*-value, *T1w/T2w* mean regional values of T1-weighted/T2-weighted, *TMT-A* part A of trail making test, *WM* white matter

We found no significant differences on ∆-MRI parameters between patients with cognitive worsening (*N* = 21) and nonworsening (*N* = 5) (*p* > 0.05).

Logistic regression analysis showed that ∆-values of WM density in the cerebellum/brainstem (*β* = 2188.70, *p* = 0.02) significantly predicted cognitive worsening at *T*_1_, explaining 64% of the variance (*R*^2^ = 0.64).

Finally, in MSA-P, significant correlations were found between ∆-values of GM density in the cerebellum/brainstem and 15-RAWLT- immediate recall (rho =  − 0.61; *p* = 0.04), between ∆-values of WM density in the cerebellum/brainstem and BJLO (rho = 0.63; *p* = 0.03), between ∆-values of T1w/T2w in the Right putamen and phonological fluency (rho = 0.78; *p* = 0.02), and between ∆-values of T1w/T2w in the Left putamen and phonological fluency (rho = 0.80; *p* = 0.02).

In MSA-C, significant correlations were found between ∆-values of GM density in the cerebellum/brainstem and nonword repetition E.N.P.A. test (rho =  − 0.59; *p* = 0.04), ∆-values of GM density right putamen and Prosa test (rho = 0.69; *p* = 0.01), ∆-values of GM density in the left putamen and non-words repetition E.N.P.A. test (rho =  − 0.72; *p* = 0.01), ∆-values of T1w/T2w in the cerebellum/brainstem GM and both ROCF recall (rho = 0.64; *p* = 0.047), and TMT-A (rho =  − 0.76; *p* = 0.01).

As for subtype analysis, no correlations survived the correction for multiple comparisons (*p* < 0.05).

## Discussion

Our findings support the hypothesis that a relationship exists between pathological changes in the putamen and cerebellum of MSA patients and cognitive deficits. We focused on putamen and cerebellum since their involvement is prominent in the pathophysiology of MSA. Besides analyzing correlations between MRI parameters and an extensive neuropsychological battery, we also focused on the predictive role of MRI changes for worsening of cognitive status that is more useful for a clinical approach.

We found that putaminal T1w/T2w ratio predicted the global cognitive status at *T*_0_. This result supports our hypothesis on the relationship between these structures and the cognitive state in MSA. Indeed, putamen is mostly involved in the motor circuit and less is known about its possible implication in cognitive functioning. It is known that putamen is involved in habits learning and the stimulus–response association and seems to facilitate learning implementation, possibly accounting for our results. In a previous study on healthy controls performing neuropsychological tests [17], functional magnetic resonance imaging (fMRI) showed putamen activation during actions that followed cognitive switches and a greater activation was observed during novel as compared to routine actions. Global cognitive status, assessed by MOCA in PD patients, was found to be related to putaminal connectivity and atrophy by fMRI [18] and VBM [19], respectively. Attentional and executive functions were related to event related potentials P300 and putaminal volume in PD patients [20]. Finally, the putamen can be involved in cognitive functioning also through the motor modality, but the distinction between cognitive and higher order motor organization can be only artificial since both cognitive and motor organization of putamen may contribute to a spectrum of functions [21].

On the other hand, the progression of cognitive deterioration appeared to be related only with WM density in the cerebellum/brainstem that accounted for 64% of variance.

To our knowledge, the association between cerebellar changes and progression of cognitive impairment in MSA has never been described previously. How the cerebellum contributes to cognitive dysfunction in neurodegenerative diseases has not been extensively analyzed, but a recent study proposed that cerebellar atrophy may be a novel imaging biomarker for predicting dementia progression in patients with amyloid-negative amnestic MCI [22]. Indeed, the cerebellum may be involved in cognition through interaction with the cerebral cortex, since it has functional connections with the prefrontal and parietal cortices, which serve higher cognitive functions [23], but further studies are needed to understand if the progression of cerebellar neurodegeneration simply parallel cognitive impairment in MSA or is involved in the physiopathology of cognitive changes in MSA. Previous evidence coming from cross-sectional studies suggest a relationship between cognition and cerebellum. Specifically, in MSA-C patients, the severity of atrophic changes in the cerebellum, thalamus, and pericalcarine gyrus were significantly correlated with attentional, executive and visuospatial dysfunctions [4]. These data are in line with our findings, although the neuropsychological batteries used in previous studies are generally different. Cerebellum has been already reported as possibly involved in cognitive deficits of MSA patients. In fact, previous studies suggest that cognitive impairment in MSA may reflect functional alterations of cerebellum, consistently with our results [8]. Indeed, cerebellum is recognized to be associated to executive, visuo-spatial, and linguistic impairment [24]. An activation of the cerebellum in cognitive tasks is described for sensory processing [25], appreciation of timed intervals [26], anticipatory planning and prediction (shifting attention tasks) [27, 28], verbal working memory [29] and mental imagery [30] tasks. In PD, a correlation between MOCA scores and cerebellum activation assessed by fMRI has been described [18]. In patients with MSA, volumetric abnormalities of the cerebellum appeared to contribute substantially to both motor and cognitive performance [31] and WM cerebellar changes were linked with global cognitive status [32]. Moreover, fMRI showed a correlation between global cognitive scores and cerebellar functional connectivity also in MSA patients [33]. More specifically, it was reported that posterior lobe integrity was positively correlated with cognitive assessment [31] and the functional connectivity between right lobules VI/crus I and medial prefrontal/anterior cingulate cortices in the cerebellum was associated with cognitive decline, in a resting state functional network analysis [33].

As for specific cognitive functions, we found that some executive/attentional tasks are significantly correlated with MRI variables, and visuo-spatial abilities and memory are also involved when considering the two MSA subtypes separately. In particular, we found that constructional apraxia at baseline was related with atrophy and altered tissue integrity in the cerebellum. The constructional apraxia is a heterogeneous construct that incorporates visuospatial, perceptive, attentive, design, and motor mechanisms [34]. Indeed, it is known that CA is underpinned by the parietal lobe with its connections to premotor and motor areas and subcortical motor structures, such as the basal ganglia and cerebellum, thus supporting our results [35].

Moreover, we found that TMT-A worsening over time was related with both changes in cerebellar WM tissue integrity and putaminal volume. The TMT-A assesses attention, visual-motor, and visual-conceptual tracking, and is a sensitive task for the early stages of cognitive deterioration [36] and dementia [37]. It is also used to assess the progression of cognitive decline and differentiate demented patients from control subjects [38, 39].

A relationship between verbal repetition tasks and atrophy of putamen was also found in our study; since there is a close relationship between repetition tests and executive functions, we suggest that this finding may be accounted for by changes in the fronto-subcortical circuits [40].

In a recent paper, Sugiyama et al. [41] studied myelin maps by the T1-weighted/T2-weighted ratio of the whole brain in MSA patients and found a significantly higher WM T1w/T2w ratio in cognitively impaired patients as compared to cognitively preserved ones. In this paper, cognitive impairment was assessed by a single scale, that is the Addenbrooke’s Cognitive Examination III, and MRI parameters were calculated for the whole brain, while we used a comprehensive neuropsychological battery and analyzed relationships between both global and specific cognitive impairment and brain regions mainly affected in MSA.

In MSA-P, we found a relationship between density and T1w/T2w of putamen and cerebellum and memory, visuo-spatial abilities, and mental flexibility in accordance with previous studies [3, 4, 31, 42]. This finding is not surprising due to the wide connections between these subcortical structures and frontal, parietal and temporal lobes [6, 43–45]. Similarly, in MSA-C, significant correlations were found between ∆-values of GM density values in the cerebellum/brainstem and repetition ability, putamen, and long-term verbal memory regarding semantically organized verbal material and repetition ability, T1w/T2w in the GM cerebellum/brainstem and spatial long-term memory and attention, visual-motor, and visual-conceptual tracking. Also, these data can be explained by the cortico-subcortical circuits [6, 43–45].

Even recognizing the short follow-up, the absence of longitudinal comparison data of healthy controls, and the small sample as possible limitations of our study, our results support the idea of a subcortical cognitive impairment in MSA and suggest a role for the putamen and the cerebellum in the cognitive changes of MSA, probably due to their connections with the cortex. The putaminal T1w/T2w ratio deserves further studies as a marker of cognitive impairment in MSA.

## Supplementary Information

Below is the link to the electronic supplementary material.

Supplementary file1 (DOCX 14 kb)

## Data Availability

The datasets generated during and/or analyzed during the current study are available from the corresponding author on reasonable request.

## Abbreviations

15-RAWLT: Rey’s auditory 15-word learning test
ADL: Based activities of daily life
BJLO: Benton’s judgment of line orientation
CA: Constructional apraxia
CDT: Clock drawing test
F: Female
FDG-PET: Fluorodeoxyglucose-positron emission tomography
GM: Gray matter
IADL: Instrumental activities of daily life
IQR: Interquartile range
M: Male
MCI: Mild cognitive impairment
MDS: Movement Disorders Society
MMSE: Mini-Mental State Examination
MOCA: Montreal Cognitive Assessment battery
MSA: Multiple system atrophy
MSA-C: Multiple system atrophy with predominant cerebellar ataxia
MSA-NC: Multiple system atrophy with normal cognition
MSA-P: Multiple system atrophy with predominant parkinsonism
MRI: Magnetic resonance imaging
*N*: Number
*p*: *p*-Value
PD: Parkinson’s disease
SPSS: Statistical Package for Social Science
TMT: Trial making test
TMT-A: Part A of Trail Making Test
*T*_0_: Baseline
*T*_1_: Follow-up 1
T1w/T2w: Mean regional values of T1-weighted/T2-weighted
UMSARS: Unified Multiple System Atrophy Rating Scale
WM: White matter
