# Supplementary material for: Magnetic Resonance T1w/T2w Ratio in the Putamen and Cerebellum as a Marker of Cognitive Impairment in MSA: a Longitudinal Study
Source: Cerebellum. 2022 Aug 19;22(5):810–7. doi: 10.1007/s12311-022-01455-8 (PMC10485110; doi:10.1007/s12311-022-01455-8)
Supplement: Supplementary file 1 — Supplementary file1 (DOCX 14 kb) [file 12311_2022_1455_MOESM1_ESM.docx]

**Supplementary Material, S1:** The percentage of complete tests at T_0_ and at T_1_

|  | **T_0_** | | | **T_1_** | | |
| --- | --- | --- | --- | --- | --- | --- |
|  | **N°** | **%** | **mean±SD** | **N°** | **%** | **mean±SD** |
| **MOCA** | 26 | 100% | 21.46±4.91 | 24 | 92,3% | 19.54±4.72 |
| **15-RAWLT-recall** | 26 | 100% | 7.5±3.62 | 24 | 92,3% | 8.21±3.15 |
| **Prosa Test** | 26 | 100% | 11.45±4.52 | 24 | 92,3% | 9.57±4.00 |
| **CA** | 26 | 100% | 11.42±4.53 | 24 | 92,3% | 9.54±2.28 |
| **BJLO** | 26 | 100% | 18.46±5.78 | 24 | 92,3% | 16.58±7.71 |
| **CDT** | 26 | 100% | 8.68±2.23 | 24 | 92,3% | 7.42±2.17 |
| **ROCF-copy** | 26 | 100% | 27.82±6.78 | 24 | 92,3% | 23.38±7.95 |
| **ROCF-recall** | 26 | 100% | 13.09±7.58 | 24 | 92,3% | 12.89±7.47 |
| **TMT A** | 26 | 100% | 75.61±36.95 | 24 | 92,3% | 82.38±46.60 |
| **Verbal fluency** | 26 | 100% | 25.25±12.75 | 24 | 92,3% | 24.79±14.87 |
| **SCWT** | 26 | 100% | 10.50±12.95 | 24 | 92,3% | 3.73±5.59 |
| **ENPA- words repetition** | 24 | 92,3% | 9.29±0.81 | 24 | 92,3% | 9.54±0.83 |
| **ENPA- non-words repetition** | 24 | 92,3% | 4.17±1.08 | 24 | 92,3% | 4.04±0.95 |
| **ENPA- auditory comprehension** | 24 | 92,3% | 13.29±1.46 | 24 | 92,3% | 13.21±1.32 |

**Abbreviations:** 15-RAWLT, Rey auditory verbal learning test; BJLO, Benton orientation line test; CA, constructional apraxia test; CDT, Clock design test; E.N.P.A., neuropsychological examination for aphasia; MOCA, Montreal Cognitive Assessment; N, number; Prosa Test, short story test; ROCF, recall and copy of Rey Osterrieth figure; SCWT, Stroop Interference Test- error effect; SD, standard deviation; T_0,_ baseline_;_ T_1_, follow up; TMT-A, Trail Making Test-part A.
